# Prostate Artery Embolization (PAE) with Small Beads for the Treatment of Benign Prostatic Hyperplasia (BPH)

**DOI:** 10.3390/jpm14060613

**Published:** 2024-06-08

**Authors:** Genti Xhepa, Lucilla Violetta Sciacqua, Andrea Vanzulli, Andrea Enzo Canì, Velio Ascenti, Alexis Ricoeur, Andrea Antonio Ianniello, Agostino Inzerillo, Paolo Nicotera, Filippo Del Grande, Anna Maria Ierardi, Gianpaolo Carrafiello

**Affiliations:** 1 Istituto di Imaging della Svizzera Italiana (IIMSI), Ente Ospedaliero Cantonale (EOC), 6900 Lugano, Switzerland; filippo.delgrande@eoc.ch; 2 Interventional Radiology Unit, University Hospital of Geneva (HUG), 1205 Geneva, Switzerland; alexis.ricoeur@hcuge.ch; 3 Postgraduate School in Radiodiagnostics, University of Milan, 20122 Milan, Italy; lucilla.sciacqua@unimi.it (L.V.S.); andrea.vanzulli@unimi.it (A.V.); velio.ascenti@unimi.it (V.A.); 4 Department of Radiology, Fondazione IRCCS Istituto Nazionale dei Tumori di Milano, 20133 Milan, Italy; 5 Department of Diagnostic and Interventional Radiology, Ospedale di Garbagnate Milanese “Guido Salvini”, ASST Rhodense, 20024 Garbagnate Milanese, Italy; aecani@asst-rhodense.it; 6 Department of Radiology, Luigi Sacco University Hospital, 20157 Milan, Italy; ianniello.andrea@asst-fbf-sacco.it; 7 AOUP Paolo Giaccone, Biomedicine, Neuroscience and Advanced Diagnostic Department (BiND), University of Palermo, 90127 Palermo, Italy; inzerilloagostino@gmail.com; 8 Radiology Unit, Ospedale di Circolo e Fondazione Macchi, University of Insubria, 21100 Varese, Italy; paolonicotera78@gmail.com; 9 Facoltà di Scienze Biomediche, Campus Est, Università Della Svizzera Italiana (USI), 6900 Lugano, Switzerland; 10 Department of Radiology, Foundation IRCCS Ca’ Granda-Ospedale Maggiore Policlinico, University of Milan, 20122 Milan, Italy; anna.ierardi@unimi.it (A.M.I.); gianpaolo.carrafiello@unimi.it (G.C.)

**Keywords:** Benign Prostatic Hyperplasia, Lower Urinary Tract Symptoms, Prostate Artery Embolization, urinary catheter, interventional radiology

## Abstract

Benign Prostatic Hyperplasia (BPH) is the most frequent cause of Lower Urinary Tract Symptoms (LUTSs) in elderly populations. Minimally invasive treatments of BPH are safe and effective and are gaining popularity among both professionals and patients. Prostate Artery Embolization (PAE) has proven to be effective in Trans-Urethral Resection of the Prostate (TURP) in terms of prostate volume reduction and LUTS relief. PAE entails the selective catheterization of the prostatic artery and later embolization of distal vessels with beads of various calibers. Universal consensus regarding the ideal particle size is yet to be defined. We retrospectively evaluated 24 consecutive patients (median age: 75 years; range: 59–86 years) treated with PAE at our institution from October 2015 to November 2022. Particles of different sizes were employed; 12 patients were treated with 40–120 µm particles, 5 with 100 µm, 5 with 100–300 µm and 2 with 250 µm. Technical success, defined as selective prostate artery catheterization and controlled release of embolizing beads, was achieved in all patients. Removal vs. retention of the urinary catheter at the first post-procedural urological visit was the main clinical objective. No major peri-procedural complications were recorded, with 56% of patients successfully removing the urinary catheter.

## 1. Introduction

Benign Prostatic Hyperplasia (BPH) represents a non-neoplastic condition characterized by aberrant cellular proliferation within the transitional zone (TZ) of the prostate gland. The escalating prevalence and incidence of BPH have been robustly associated with advancing age demographics [1]. Recent scholarly inquiries have delineated a panoply of modifiable risk factors intricately involved in the genesis of early-stage BPH. These encompass serum dihydrotestosterone (DHT) concentrations, adiposity indices, glucose homeostasis perturbations, dietary constituents, physical activity regimens, and inflammatory mediators [1,2,3,4].

Autopsy-derived epidemiological data delineate a progressive increase in the prevalence of BPH with advancing age, standing at 8% in the fourth decade, escalating to 50% in the sixth decade, and peaking at 80% in the ninth decade [1]. This condition presents formidable clinical challenges, particularly as it predominantly afflicts elderly individuals burdened with a plethora of coexisting medical conditions [1]. BPH emerges as the principal instigator of Lower Urinary Tract Symptoms (LUTSs), afflicting an estimated 70% of males beyond the age of 80 [5]. LUTS, characterized by their incapacitating nature, exert a significant toll on the quality of life for those affected. The assessment of LUTS impact commonly relies on the International Prostate Symptom Score (IPSS), a validated tool employed to discern the extent of symptomatology’s intrusion into daily activities [6].

Acute Urinary Retention (AUR) stands out as the most critical complication associated with BPH, constituting a life-threatening event that ensues in up to 10% of men aged 70–79 diagnosed with BPH [7,8].

AUR mandates immediate intervention to alleviate obstruction, typically through catheterization, as its unmitigated progression poses a significant risk of precipitating acute renal injury [9,10]. Symptomatic BPH engenders a diverse array of therapeutic strategies for effective management.

In accordance with the guidelines set forth by the European Association of Urology (EAU), initial interventions for LUTS attributed to BPH predominantly center on medical interventions and lifestyle adjustments [11]. Implementation of straightforward behavioral modifications, such as restricting evening fluid intake, holds promise in attenuating the frequency of nocturia. Additionally, the avoidance of diuretic agents and incorporation of pelvic floor exercises present viable strategies for mitigating urinary frequency, a pivotal component of the IPSS [11].

The utilization of alpha 1-adrenoceptor antagonists (α1-blockers), either as monotherapy or in conjunction with 5-alpha reductase inhibitors (5-ARIs), stands as a cornerstone in alleviating BPH-related symptoms [12]. However, in instances where primary interventions prove inadequate or elicit intolerable adverse effects, surgical intervention becomes imperative.

According to the latest guidelines, transurethral resection of the prostate (TURP) remains the gold standard surgical intervention for managing BPH [13,14,15]. TURP involves the meticulous insertion of a resectoscope through the penile urethra, targeting the central prostatic region responsible for symptomatic presentation. This procedure aims to effectuate rapid de-obstruction. Both bipolar (b-TURP) and monopolar (m-TURP) techniques are commonly employed and yield comparable outcomes in terms of symptom relief. However, b-TURP often demonstrates superior efficacy and is associated with lower complication rates, particularly in cases involving larger prostates [16,17].

Key complications reported with TURP encompass clot retention (2–5%), necessitating post-procedure blood transfusions (0.4–7.1%), urinary tract infections (1.7–8.2%), and less frequent occurrences of TUR syndrome (0–1.1%). TUR syndrome arises from the absorption of electrolyte-free irrigating fluid and represents a potentially serious complication of the procedure [18,19,20,21].

Notwithstanding the considerable success of TURP in managing BPH, the potential for complications or contraindications has instigated the exploration of innovative, minimally invasive therapeutic avenues.

The introduction of laser-based prostate enucleation techniques has emerged as a significant development, notably featuring holmium laser enucleation of the prostate (HoLEP) and thulium laser enucleation of the prostate (ThuLEP) as prominent modalities [18]. Comparative investigations between HoLEP and TURP have underscored several advantages associated with HoLEP, including abbreviated hospitalization durations, reduced periods of catheterization, and augmented hemostatic capabilities, thereby mitigating bleeding-related complications. However, HoLEP typically entails a lengthier operative duration, primarily attributable to the additional morcellation time necessitated [22,23,24,25,26,27].

Within the realm of Minimally Invasive Surgical Therapies (MISTs), Prostatic Urethral Lift (PUL) and Water Vapor Thermal Therapy (WVTT), also known as Rezum, have garnered attention as promising alternatives, exhibiting notable efficacy in ameliorating urinary symptoms among treated individuals [28,29]. The PUL technique involves the deployment of the UroLift Device, which releases small permanent implants to mechanically widen the prostatic urethra, thereby alleviating symptomatic obstruction [30]. Conversely, WVTT represents an outpatient procedure leveraging radiofrequency technology. Here, a transurethral device administers water steam to the prostate’s transition zone, inducing coagulative necrosis within the treated area [31,32].

Innovative minimally invasive techniques have emerged as pivotal adjuncts in the therapeutic armamentarium for BPH, encompassing ablative methodologies such as Transurethral Needle Ablation (TUNA), Transurethral Microwave Ablation (TUMT), and partial cryoablation [33,34,35,36,37,38]. Following the insertion of specialized devices into the urethra, these techniques achieve coagulative necrosis of prostatic tissue, employing either radiofrequencies (as in TUNA) or microwaves (as in TUMT).

The absence of a universally superior minimally invasive modality underscores the complexity of BPH management [15,16,31]. Factors including prostate volume, risk of sexual dysfunction, surgical risk, and patient preferences collectively inform the selection of the most appropriate treatment modality for individual patients [16,18].

According to the Society of Interventional Radiology (SIR), Prostate Artery Embolization (PAE) represents a viable therapeutic avenue for addressing LUTS attributed to BPH, exhibiting comparable efficacy to surgical interventions [16,39,40,41,42]. The allure of PAE is further accentuated by its associated benefits, including reduced hospitalization durations, diminished transfusion risks, and a low incidence of sexual dysfunction [16,41,43].

Initially relegated to emergency scenarios such as hemorrhage, PAE has demonstrated remarkable efficacy in reducing prostate volume, albeit with delayed onset of action and fewer procedure-related risks compared to surgical interventions [44]. Presently, PAE emerges as an optimal strategy for patients unsuitable for surgery, those averse to surgical interventions, or individuals prioritizing the preservation of sexual function [16,40].

The primary risk associated with PAE pertains to non-target embolization, with unfavorable vascular anatomy serving as a principal contraindication to the procedure [43]. Selective catheterization of prostate arteries is facilitated through microcatheters or microguides, followed by occlusion employing various embolic agents. Spherical polyvinyl alcohol (PVA) particles of varying diameters, notably within the ranges of 100–300 μm, 250–400 μm, or 300–500 μm, constitute the predominant embolic agents employed [45,46,47]. The optimal bead caliber remains a subject of ongoing discourse [48,49].

In the study conducted by Bilhim et al., a meticulous examination of the embolic particle size’s impact on the efficacy and safety profile of PAE was undertaken [49]. Their findings unveiled a nuanced interplay between particle size and clinical outcomes, delineating distinct advantages and limitations associated with different particle sizes.

Notably, larger embolic particles (>200 μm) exhibited superior clinical outcomes in subjective parameters such as the International Prostate Symptom Score (IPSS) and peak flow rate (Qmax) [49]. Conversely, smaller particles demonstrated enhanced efficacy in objective parameters, particularly in reducing Prostate-Specific Antigen (PSA) levels [49]. This dichotomy in efficacy can be attributed to the deeper penetration of smaller particles into the distal prostatic arteries, inducing a more extensive area of ischemia and subsequent prostate shrinkage.

However, this apparent advantage of smaller particles in reducing prostate volume was tempered by concerns regarding increased side effects reported in some early studies [47]. This phenomenon was hypothesized to result from the deeper penetrating nature of smaller particles, potentially predisposing them to enter anastomotic arterial vessels and lead to non-target embolization.

Nevertheless, Bilhim et al.’s investigation provided reassurance by demonstrating that there were no significant differences in adverse events following PAE with 100 μm or 200 μm PVA particles [49]. This observation suggests a potential equilibrium between therapeutic efficacy and safety across different particle sizes.

In our own study, we aimed to extend these findings by evaluating the response to PAE treatment utilizing particles of varying diameters in a cohort of patients affected by LUTSs attributable to BPH. Additionally, we sought to comprehensively analyze the occurrence of potential complications associated with different particle sizes. 

Our assessment of treatment response was predicated on the retention or removal of the bladder catheter, serving as a clinically relevant indicator of treatment efficacy and patient comfort.

## 2. Materials and Methods

In our retrospective study, we analyzed a cohort consisting of 24 consecutive patients, with a median age of 75 years (range: 59–86 years), who underwent PAE utilizing beads of various sizes (ranging from 40 to 300 μm) at our medical institution between October 2015 and November 2022.

Before the initiation of treatment, the cohort exhibited an average prostate volume of 116.5 mL, with prostate volumes ranging from 26 mL in cases previously managed with TURP to a maximum of 309 mL. Notably, all patients had a dependency on indwelling urinary catheters due to antecedent episodes of AUR attributed to the presence of large central adenomas. Additionally, one patient presented with persistent hematuria.

Inclusion criteria for enrollment in this study were based on the absence of prostate cancer or other neoplastic pathologies, favorable anatomical suitability for PAE, and documented failure of initial-line therapeutic interventions, as delineated in Table 1.

Two patients subjected to PAE had previously undergone surgical intervention via TURP, but experienced a recurrence of AUR necessitating catheter repositioning. Intraoperative assessment of vascular anatomy feasibility was conducted via arteriography.

Exclusion criteria were applied to the following three patients: one presenting with synchronous bladder cancer, another necessitating coil deployment instead of beads to address a bleeding pseudoaneurysm, and a third case in which embolization was aborted due to the presence of a contraindication (penile anastomosis) detected during intraprocedural imaging. Additionally, two patients were excluded due to PAE procedures utilizing both small and large particles (one treated with 100–300 µm and 40–120 µm, and the other with 100 µm and 300–500 µm particles).

**Figure 1:**
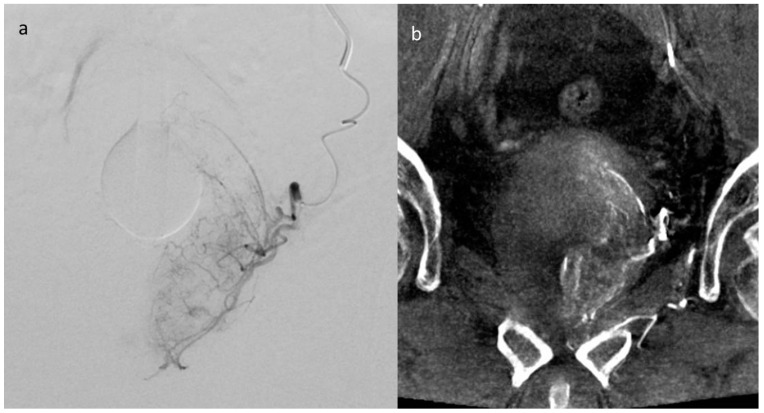
(**a**) Superselective Digital Subtraction Angiography (DSA) and (**b**) Cone Beam Computed Tomographic Angiography (CBCTA) of the left prostate artery.

In all instances, super-selective catheterization of prostatic arteries was meticulously performed using microcatheters to minimize the risk of non-target embolization. The correct positioning of microcatheters was meticulously verified utilizing Cone Beam Computed Tomographic Angiography (CBCTA), as exemplified in Figure 1.

PAE was performed under local anesthesia using the unilateral approach in 8 patients or the bilateral femoral approach in 16 patients, using particles with different sizes in a range between 40 µm and 300 µm; 12 patients were treated with 40–120 µm particles, 5 patients with 100 µm, 5 patients with 100–300 µm and 2 patients with 250 µm. 

## 3. Results

Technical success, defined as super-selective catheterization of the prostate artery and the controlled release of embolizing beads, was achieved in all patients. For the evaluation of complications, we considered the latest updated classification of adverse events (AEs) proposed by the Society of Interventional Radiology (SIR) [50,51]. Considering every scenario contemplated by the SIR, no major procedure-related complications were registered. Additionally, there were no cases of increased hospitalization.

The clinical evaluation was performed for only a few patients by using the IPSS questionnaire, as most of them were outpatients, followed outside the hospital. Therefore, we did not take the IPSS into consideration in the final evaluation of our work.

The primary clinical outcome measured was the removal of the urinary catheter at the first post-procedural follow-up.

At follow-up intervals of 6 months and 1 year, the outcomes related to urinary catheter use were assessed across all patients, irrespective of the particle size used in the procedure. Only one patient did not require catheter placement immediately after the procedure due to effective urination with no post-void residuals (PVRs). In contrast, 56% of patients were able to remove the catheter at their first post-procedural visit, even when presenting with minimal or non-pathological PVR. However, one patient required catheter repositioning one month after the procedure due to a relapse of AUR. Additionally, 20% of patients were unable to remove the catheter after PAE due to high PVR levels.

When analyzing outcomes based on the size of the embolizing particles, the following observations were made: among the twelve patients treated with the smallest particles (40–120 µm), one patient did not require bladder catheterization, nine patients removed the catheter at the first post-procedural visit, and one patient needed catheter repositioning one month after PAE due to recurrence of AUR.

Among the five patients treated with 100 µm particles, two were successful in removing the catheter, two required continued catheter use, and one patient needed a ureterocutaneostomy due to persistent hematuria, which was unrelated to the procedure.

Of the five patients treated with 100–300 µm particles, three were able to remove the catheter at the first post-procedural visit, while two needed to maintain catheter use.

Patients treated with 250 µm particles were all unsuccessful in removing the catheter following the procedure.

A comparative analysis of patient outcomes based on the size of embolizing particles used during PAE revealed significant differences in the success rates of catheter removal. In the group treated with smaller particles (40–120 µm and 100 µm), 70.6% of patients were able to successfully remove their catheter. Conversely, in the group treated with larger particles (100–300 µm and 250 µm), only 42.9% of patients achieved successful catheter removal. Furthermore, patients who were able to remove their catheters did not exhibit any PVR or pathological urinary residue. 

Notably, all patients who successfully removed their catheters after the procedure did not experience any recurrence of AUR at the 1-year follow-up mark.

PAE results with different particle sizes are presented in Table 2.

In assessing prostate size before and after treatment, data from 17 cases were analyzed to derive prostate volumes at either the 6-month or 1-year post-operative follow-up. Across the cohort, a consistent decrease in gland volume was observed, except for one patient treated with small particles (40–120 µm) who exhibited no change in prostate volume.

When examining the total number of patients treated with small particles (diameters 40–120 µm and 100 µm), an average reduction in prostate volume of 76% was noted. This reduction was comparable to the average reduction of 75.6% observed among patients undergoing PAE with larger particles (100–300 µm and 250 µm).

Despite the differences in patient group sizes and the lack of statistical significance, it is noteworthy that the average post-procedural reduction in prostate volume was similar between the two groups. This observation suggests a consistent efficacy of PAE across varying particle sizes, albeit with variations in individual patient responses.

## 4. Discussion

As global life expectancy continues its upward trajectory, the prevalence of LUTS attributed to BPH escalates, especially among men aged 60 and above. These symptoms, often severe and refractory to initial pharmacological interventions, present formidable clinical challenges. Despite optimized medical management, a significant subset of patients continues to experience persistent LUTS, necessitating more invasive interventions [1]. Transurethral resection of the prostate (TURP) remains the gold standard for such cases, yet it carries inherent risks, including intra- and post-procedural bleeding, infection, and the potential for TUR syndrome [15,18,21]. However, certain patients pose complexities due to the presence of multiple comorbidities or contraindications to surgery, prompting the exploration of alternative interventions that offer comparable efficacy to TURP but with reduced invasiveness.

The clinical dilemma is particularly acute when dealing with prostates exceeding 100 mL in volume, as the risks associated with TURP, such as heightened intraoperative and perioperative complications and extended recovery periods, become magnified [52]. In response to these challenges, modalities utilizing holmium (HoLEP) or thulium (ThuLEP) lasers have emerged as promising alternatives. These techniques have demonstrated substantial efficacy in reducing prostate volume while conferring several advantages over conventional TURP, including shorter hospital stays, improved hemostasis, and lower complication rates [22,23,27].

As such, there is a burgeoning interest within the medical community in exploring these less invasive approaches as viable strategies for managing BPH-related LUTS, particularly among patient populations with heightened surgical risks or preferences for minimally invasive interventions. This underscores the imperative for further research and clinical evaluation to ascertain the optimal treatment paradigm for this challenging patient cohort.

The PUL technique has emerged as a subject of considerable interest owing to its notable efficacy, particularly in addressing the intricate challenges presented by larger prostatic volumes. This innovative approach involves the strategic application of tension to the prostatic lobes, thereby inducing compression of the prostatic urethra [30,53].

It has been demonstrating that WVTT, also known as Rezum, is very effective in IPSS improvement and it has shown a significant decrease in the bladder outlet obstruction index (BOOI). Both young and elderly patients can benefit from this technique because of its short operative time [54]. Moreover, ongoing research endeavors underscore the burgeoning evidence supporting the efficacy of WVTT in ameliorating LUTS associated with prostates exceeding the volumetric threshold of 80 cm^3^ [55]. 

Furthermore, laser-based modalities have demonstrated noteworthy efficacy in the management of larger prostatic volumes [56].

In stark contrast, ablative interventions such as TUNA and TUMT offer immediate reductions in prostatic volume. However, their efficacy tends to diminish when confronted with larger prostates [16,52]. 

Conversely, PAE has emerged as a compelling therapeutic avenue for the management of LUTS attributed to BPH. PAE not only yields outcomes akin to those achieved through TURP but also mitigates operative risks [39,40,41,42]. A salient advantage of PAE is its adaptability to prostates exceeding the volumetric threshold of 100 mL, with consistently favorable clinical outcomes [57].

The main controversy remains in the choice of embolizing particle size [48,49,58].

Some studies support that large particles are more effective in clinical improvement as assessed by IPSS, while smaller particles act more markedly on the reduction in objective parameters, such as PSA [49].

Regarding adverse events, Bilhim et al. demonstrated that there are no significant differences in adverse events after PAE with 100 μm or 200 μm PVA particles, while Wang et al. stated that there are no significant differences between the use of 50–100 μm particles or 100 μm PVA particles alone [49,58].

The central point of contention within the field of PAE revolves around the selection of embolizing particle size, a topic extensively scrutinized in the literature [48,49,58]. Various studies have contributed to this discourse, presenting divergent perspectives on the efficacy of different particle sizes in achieving clinical improvement and mitigating adverse events.

One line of inquiry suggests that larger embolizing particles may yield superior clinical outcomes, particularly evident in the amelioration of symptoms as assessed by the IPSS [49]. Conversely, smaller particles appear to exert a more pronounced effect on objective parameters such as PSA levels, thus highlighting potential nuances in the therapeutic mechanisms of different particle sizes [49].

The assessment of adverse events further complicates this matter, with studies yielding conflicting findings regarding the comparative safety profiles of different particle sizes. For instance, Bilhim et al. found no significant disparities in adverse event rates following PAE with either 100 μm or 200 μm polyvinyl alcohol (PVA) particles [49]. In contrast, Wang et al. reported similar outcomes between the use of 50–100 μm particles and 100 μm PVA particles alone [58]. These contrasting findings underscore the complexity of the relationship between particle size and procedural safety, necessitating further investigation to elucidate optimal strategies for minimizing adverse events during PAE.

Our study provides detailed insights into the outcomes of prostatic artery embolization (PAE), particularly concerning the influence of embolizing particle size. We achieved a high rate of technical success, with all patients undergoing super-selective catheterization of the prostate artery and controlled release of embolizing beads. This success was observed across all patients, regardless of the embolizing particle size used during the procedure. Furthermore, no major procedure-related complications according to the SIR Classification were noted in any of the patients, aligning with previous research indicating PAE as a safe and effective procedure for managing BPH [50,51].

In terms of clinical outcomes, our study revealed variations in catheter removal success rates following PAE. Among the patients treated with smaller particles (40–120 µm and 100 µm), a higher proportion successfully removed the catheter compared to those treated with larger particles (100–300 µm and 250 µm). Specifically, 70.6% of patients treated with smaller particles successfully removed their catheters, while only 42.9% of patients treated with larger particles achieved successful catheter removal. Notably, the success rates varied depending on the embolizing particle size used, suggesting a potential influence of particle size on clinical outcomes such as catheter removal success.

When comparing our findings to existing literature, we observed conflicting evidence regarding adverse event rates associated with different particle sizes in PAE. Bilhim et al. found no significant differences in adverse event rates between procedures utilizing 100 µm and 200 µm polyvinyl alcohol (PVA) particles [49], while Wang et al. reported similar outcomes between procedures utilizing 50–100 µm particles and those utilizing 100 µm PVA particles alone [58]. These discrepancies underscore the complexity of the relationship between particle size and procedural safety, highlighting the need for further investigation to elucidate optimal strategies for minimizing adverse events during PAE.

While our study sheds some light on the outcomes of PAE, there are several limitations that need to be acknowledged.

Firstly, the study’s sample size was relatively small, potentially limiting the generalizability of the findings. Secondly, the retrospective design of the study introduces inherent biases and limitations associated with retrospective data collection and analysis. Additionally, the study may be subject to selection bias, as patients were not randomized to different embolizing particle size groups and it was conducted at a single center. Factors influencing the choice of particle size may have confounded the observed outcomes. The follow-up period in our study was relatively short-term, with outcomes assessed at 6 months and 1 year after the procedure. Moreover, the inclusion of patients treated with a range of embolizing particle sizes introduces variability in outcomes. 

Lastly, while our study analyzed outcomes based on embolizing particle size, it did not directly compare the efficacy and safety of different particle sizes to the small sample size. 

## 5. Conclusions

In conclusion, our study underscores the significance of prostatic artery embolization (PAE) as a viable treatment option for Benign Prostatic Hyperplasia (BPH). While achieving technical success and favorable outcomes in catheter removal, our findings highlight the importance of individualizing treatment approaches based on embolizing particle size. The results of our study emphasise that small particles are very effective in reducing prostate volume and thus BPH-related LUTS with few side effects. However, the study’s limitations, including sample size constraints and retrospective design, necessitate cautious interpretation of the results. Moving forward, further research efforts should prioritize larger, prospective studies to validate our findings and optimize patient care in BPH management. Despite these limitations, our study adds valuable insights to the existing literature, reaffirming PAE’s role as a safe and effective minimally invasive intervention for patients with BPH.

## Figures and Tables

**Table 1:** Inclusion and exclusion criteria for patients receiving PAE in our study.

Exclusion Criteria	Inclusion Criteria
Prostate cancer	Favourable anatomy
Synchronous tumours	Failure of first-line therapy
Pseudoaneurysm	Presence of permanent catheter
Penile anastomosis	Contraindications to TURP
	Patients’ choice

**Table 2:** Results in terms of CV removal–retention and PVR of patients treated with PAE with beads stratified by size.

Patient#	Particles Caliber	Access	Outcome	PVR
1	40–120	Bilat	No CV	No
2	40–120	R	Removal CV	No
3	40–120	Bilat	Removal CV	130 mL
4	40–120	Bilat	Removal CV	Non-pathological
5	40–120	Bilat	Removal CV	Non-pathological
6	40–120	Bilat	Removal CV	No
7	40–120	Bilat	Removal CV	No
8	40–120	L	Removal CV	Non-pathological
9	40–120	Bilat	Removal CV	No
10	40–120	Bilat	Removal CV	No
11	40–120	Bilat	Repositioning after 1 month	Relapse of AUR
12	40–120	L	Removal CV	102 mL
13	100	R	Removal CV	No
14	100	L	Removal CV	No
15	100	Bilat	Retention of CV	High
16	100	R	Retention of CV	High
17	100	L	Ureterocutaneostomy	Haematuria
18	100–300	Bilat	Removal CV	No
19	100–300	Bilat	Removal CV	No
20	100–300	Bilat	Removal CV	No
21	100–300	L	Retention of CV	High
22	100–300	Bilat	Retention of CV	High
23	250	Bilat	Retention of CV	High
24	250	Bilat	Retention of CV	Haematuria

## Data Availability

All data are available.
